# Supplementary material for: Impact-based forecasting of tropical cyclone-related human displacement to support anticipatory action
Source: Nat Commun. 2024 Oct 10;15:8795. doi: 10.1038/s41467-024-53200-w (PMC11467304; doi:10.1038/s41467-024-53200-w)
Supplement: Supplementary file 1 — Supplementary Information [file 41467_2024_53200_MOESM1_ESM.pdf]

Supplementary Information

S1 Regional impact functions calibration for internal displacement

Here we show the grouping of the TC-prone countries with similar vulnerability. In total, there are 394 displacement events all around the globe divided into the 10 regions depicted in figure S1, with the reported number varies from a few to millions [1].

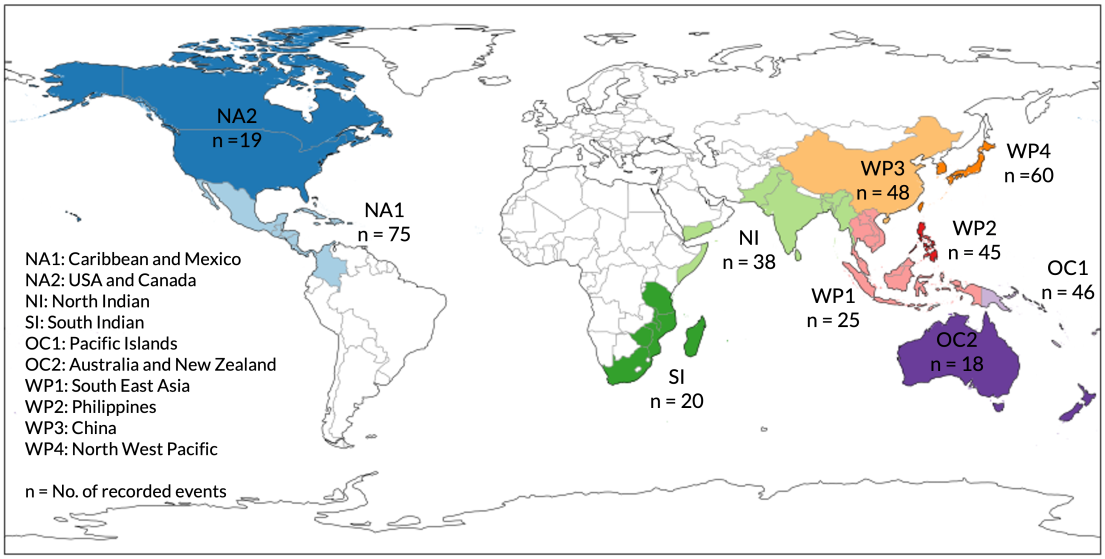

Figure S1: World map of the countries used for the impact function calibration, colour coded per calibration region. The number of resulting matched events N is displayed per region.

We assume the displacement impact functions follow a candidate sigmoidal impact function as proposed by Emanuel [2],

$$f(v) = \frac{v^3}{1+v^3}, \tag{1}$$

where v at a given location x is defined as

$$v(x) = \frac{\max\{V(x) - V_{\text{thresh}}, 0\}}{V_{\text{half}} - V_{\text{thresh}}}, \tag{2}$$

where the 1 minute sustained maximum wind speed is $V(x)$, the minimum wind speed when displacement starts to occur is parameter V_{thresh}, and the wind speed at which half of the total impact occurs is parameter V_{half}. We set $V_{\text{thresh}} = 25.7$ m/s and only estimate V_{half} in the calibration, following Eberenz *et al.* [3].

We optimise the single impact functions per region by minimising a cost function that is expressed as root-mean-squared fraction (RMSF),

$$\text{RMSF} = \exp\left(\sqrt{\frac{1}{N}\sum_{i=1}^{N}\left[\ln\frac{\hat{y}_i}{y_i}\right]^2}\right), \tag{3}$$

where N is the total number of recorded events in each region, $\hat{y}_i$ is the model estimated displacement of event i and y_i is the respective reported displacement from IDMC.

Figure S2 shows the calibration results for the RMSF optimised impact function for the 10 regions (black solid lines).

We further calibrate one impact function for each single event and take the set of functions that lies between the 10th and the 90th percentile of the resulting parameter distribution for each region (light blue lines in figure S2). This bundle of functions is used as the uncertainty distribution for the uncertainty and sensitivity analysis.

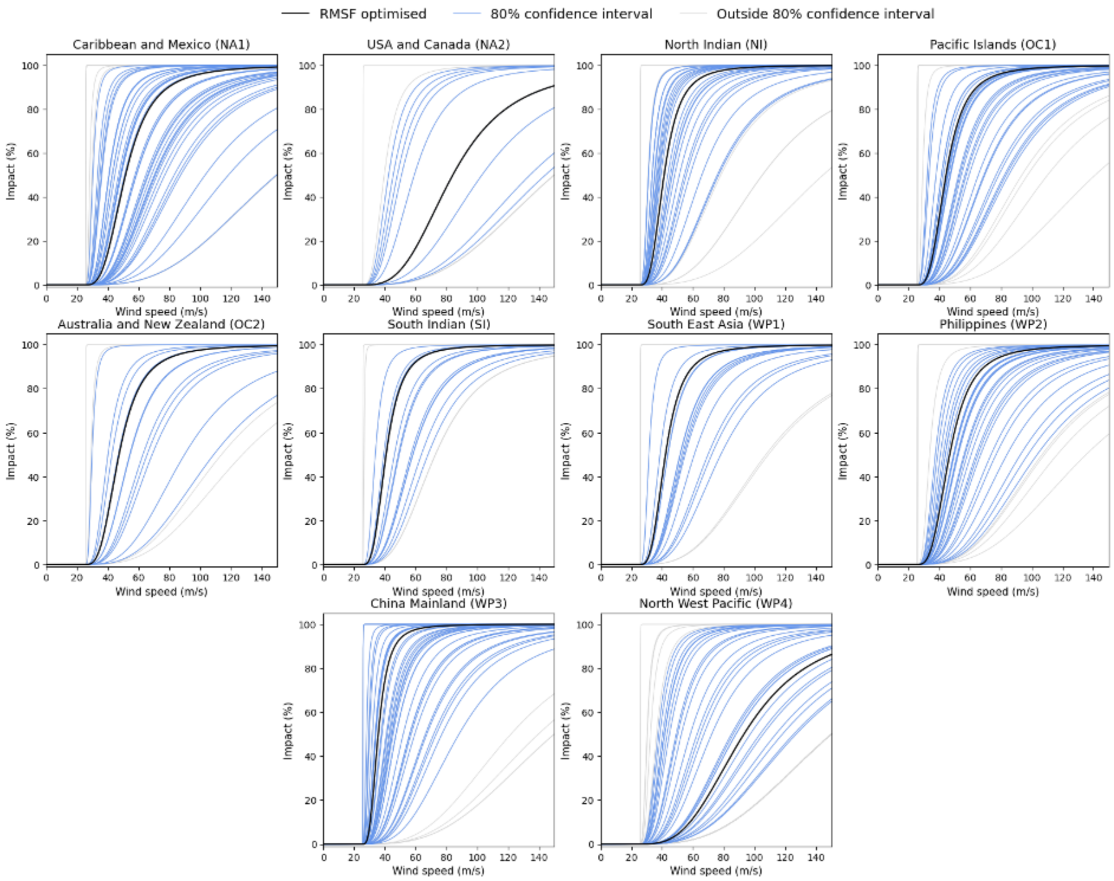

Figure S2: Calibrated regional impact functions for human displacement in 10 regions: root-mean-squared fraction (RMSF) optimised function (black lines), and the set of best-fit functions for each individual TC event in each region that lies within the 80% confidence interval, used as the uncertain inputs for the global uncertainty and sensitivity analysis (light blue lines). Grey lines show the remaining impact functions that are outside the 80% confidence interval.

S2 Impact forecast for TC Yasa at different lead time

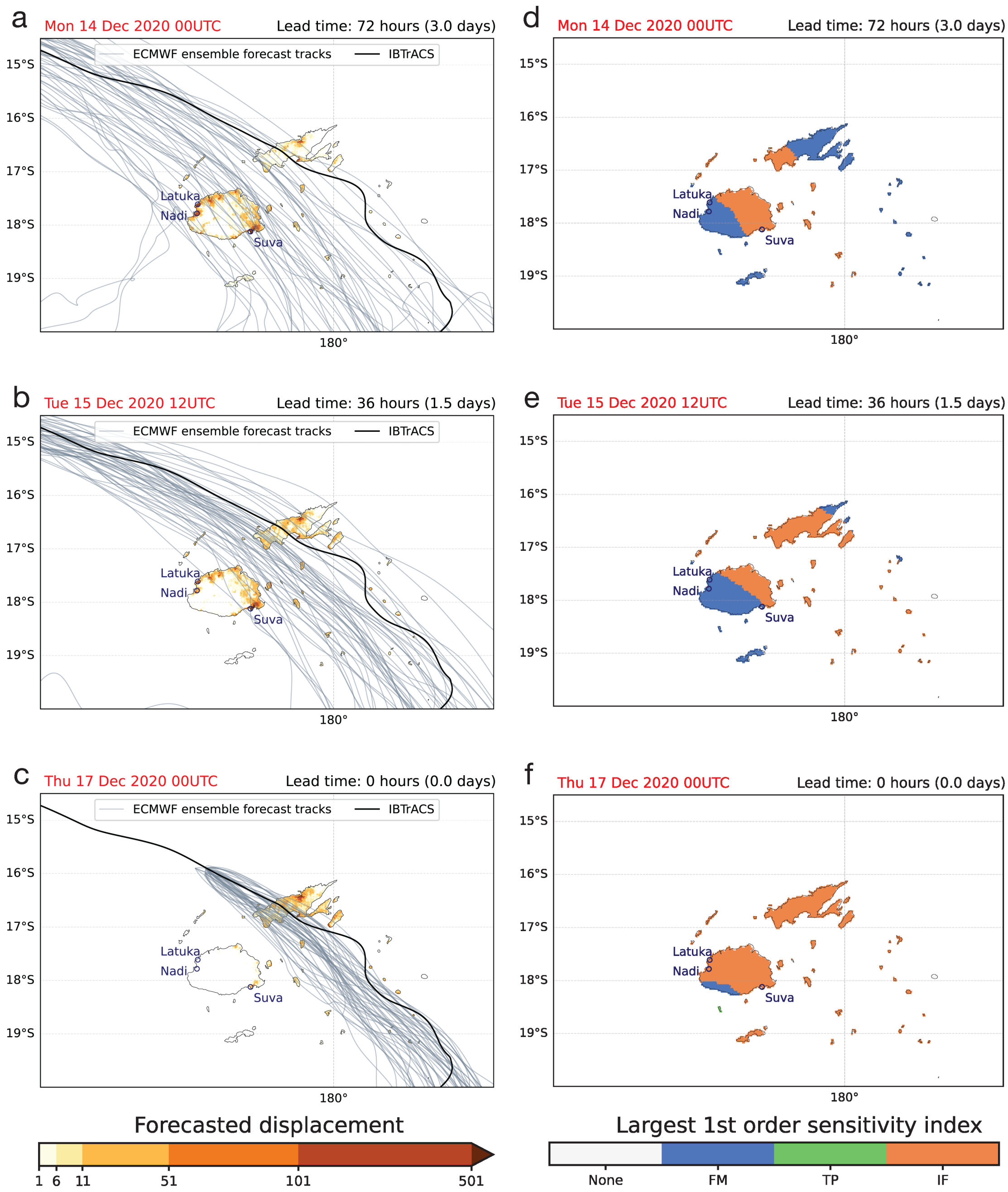

Figure S3: (a-c) The forecast-ensemble-averaged impact map of displacement by TC Yasa overlaid with ECMWF forecast tracks (grey lines) and the actual track from IBTrACS (black line; [4]) at 3-, 1.5-, and 0- days lead time, respectively. (d-f) The largest Sobol first-order sensitivity indices at each grid point, corresponding to different lead time forecast from (a-c). FM, TP, and IF denote the uncertain input parameters: forecast members, total population, and impact function, respectively.

S3 Case study: TC Harold in Vanuatu

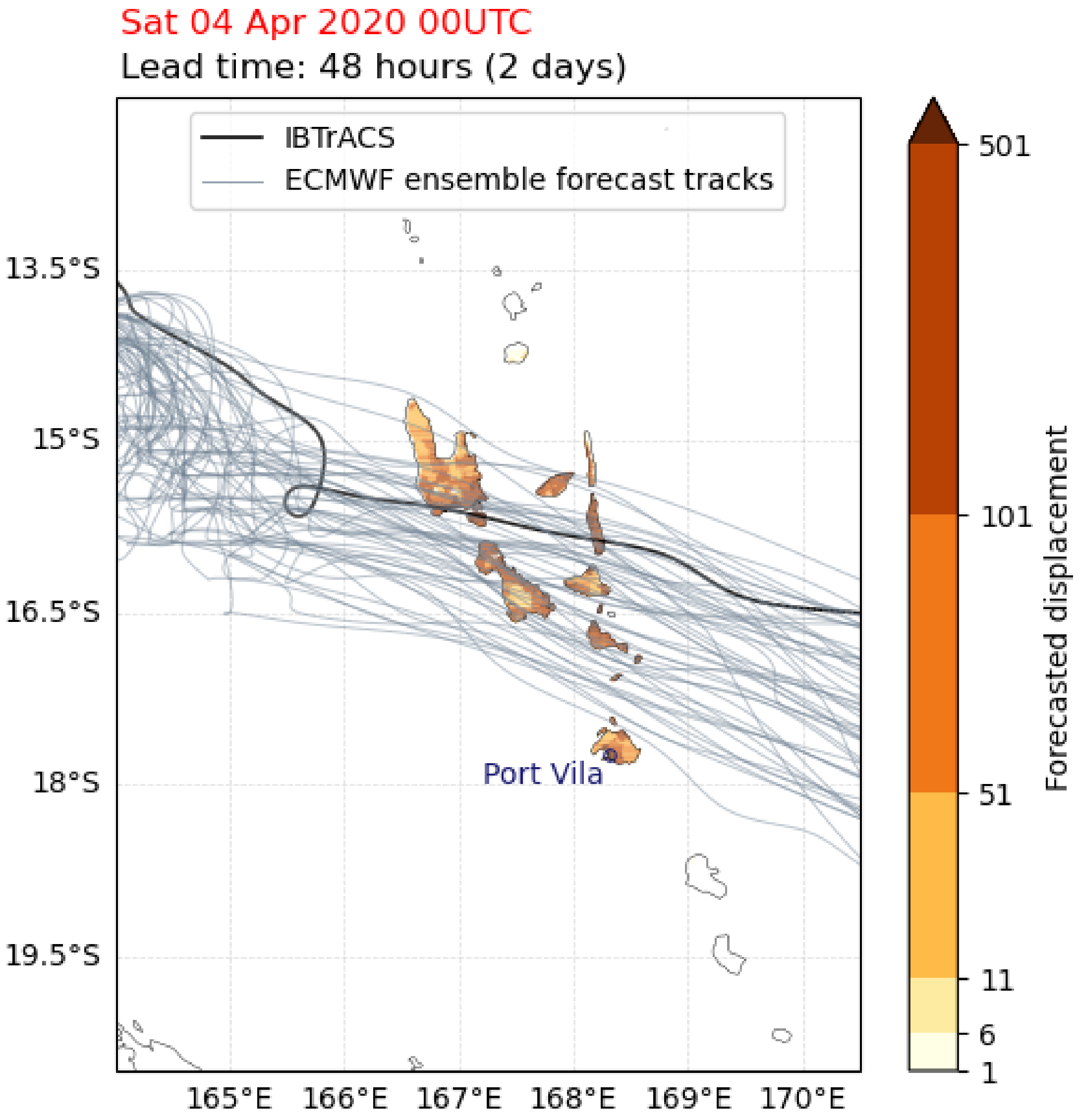

Figure S4: (a) The forecast-ensemble-averaged impact map of displacement by TC Harold in Vanuatu as forecasted at 00:00 UTC on 04 April 2020, two days before the TC landfall. The black line shows the observed best track of TC Harold from IBTrACS [4]. Grey lines show the ensemble of ECMWF forecasted TC tracks.

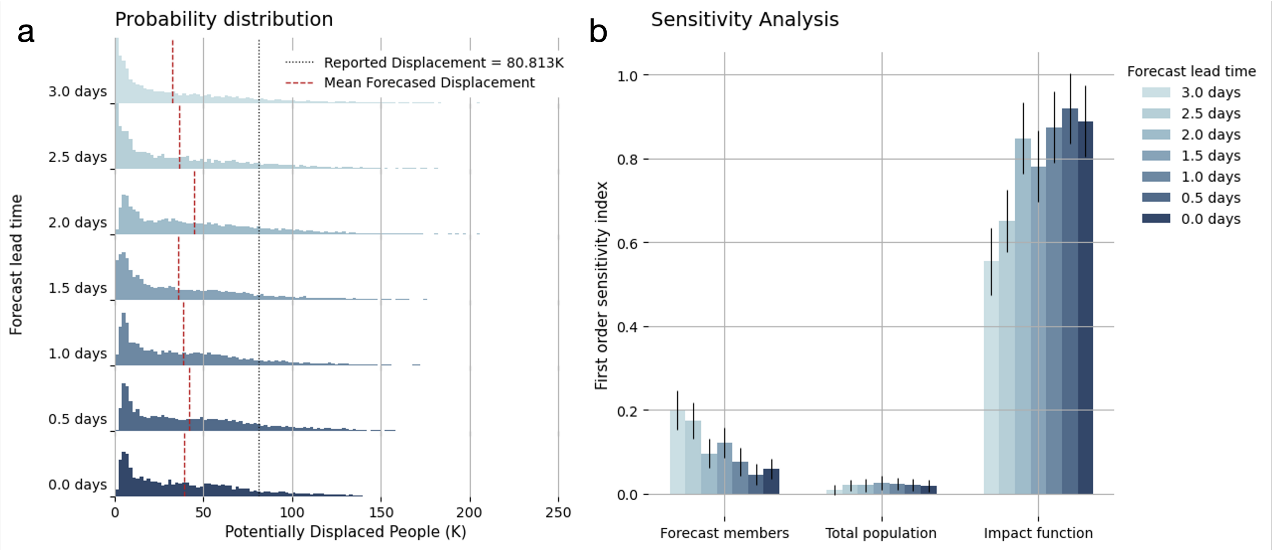

Figure S5: (a) Similar to Figure 4, probability distribution of the impact forecast and (b) first-order sensitivity indices of the different uncertainty parameters for the total number of displaced people at different forecast lead times ranging from 3 days to 0 days from the landfall of TC Harold at Vanuatu.

S4 Impact forecast validation and biases

We validate the displacement impact forecast for TC events between 2017 and 2020, where displacement was reported in the IDMC displacement database [1]. Figure S6a shows the scatter plot of the average forecasted total number of displacements from the global uncertainty analysis compared to the reported number of displacements. The plot indicates an overestimation of the forecasted number of displacements. This overestimation also exists when compared to the modelled number of displacements using the best observed tracks from IBTrACS [4].

While we calibrate the impact functions using TC tracks from IBTrACS (see section S1), we compare the TC hazard intensity from the ECMWF ensemble forecast and IBTrACS in terms of central pressure and maximum sustained wind at 10 meters above the surface. We find systematic biases indicating an underestimation (higher central pressure and lower wind speed) of the TC intensity (figure S7), consistent with findings from other studies such as Chan *et al.* [5] and Aijaz *et al.* [6]. Although the underestimation of TC hazard intensities contributes to the underestimation of the impacts, this effect is compensated by the larger wind footprints on land from the ECMWF TC forecast simulations. Figure S8 shows the number of centroids on land from the ECMWF forecast where the wind speed exceeds 25.7 m/s (the threshold where displacement starts to occur) compared to the corresponding numbers from IBTrACS. One possible explanation is that the ECMWF TC forecast (and numerical weather predictions in general) does not distinguish the transition of tropical cyclones to extra-tropical cyclones [7], while IBTrACS only records tracks of tropical cyclones [4]. Note that we do not include any bias corrections in this study, but this could be addressed in future iterations of the model.

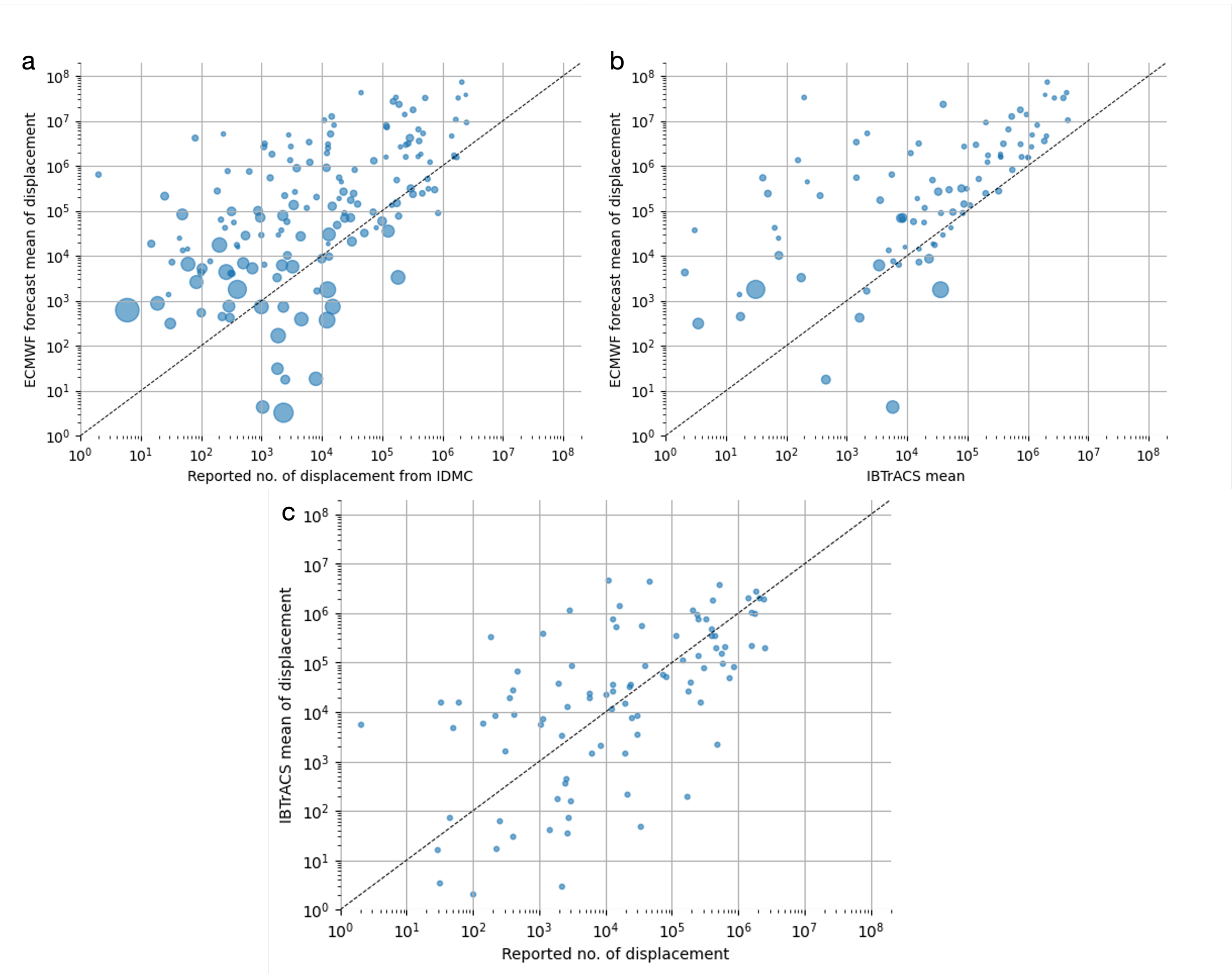

Figure S6: Scatter plot comparing the ECMWF forecast mean of displacement at 0.5 day lead time from the global uncertainty analysis and the IBTrACS mean. The size of the circles denotes the scale of the normalised standard deviations of the distribution from the probability distribution of the impact forecast.

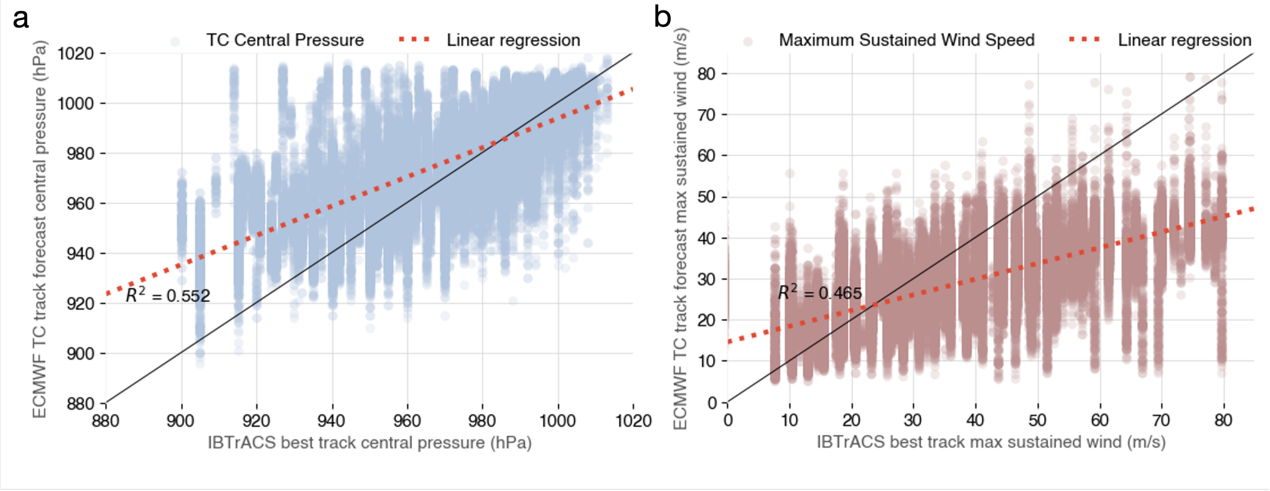

Figure S7: (a) Scatter plot comparing the central pressure of tropical cyclones (TCs) with reported displacements by IDMC between 2017 and 2020. The plot contrasts ECMWF forecast tracks with IBTrACS observed best tracks. (b) Similar to (a), but comparing maximum sustained wind at 10 metres above the surface.

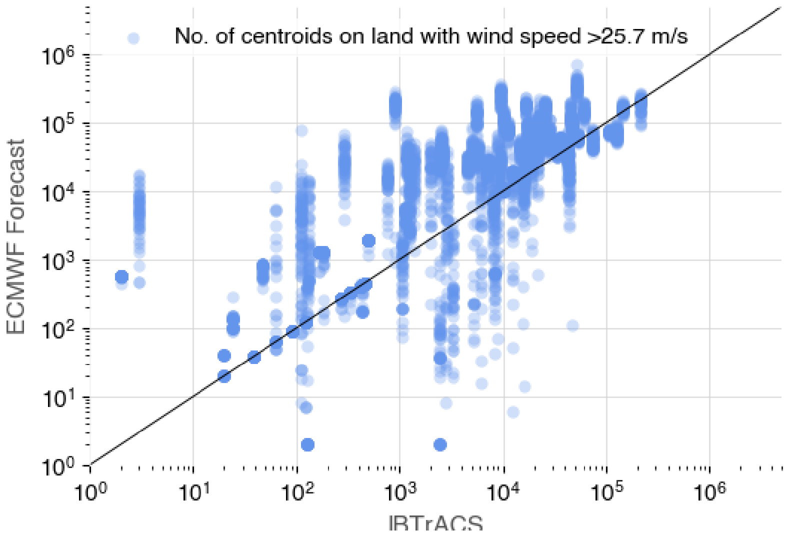

Figure S8: Scatter plot comparing the ECMWF forecast number of centroids on land with wind speeds greater than 25.7 m/s (threshold where displacement starts to occur) to the corresponding numbers from IBTrACS.

References

1. IDMC. *Global Internal Displacement Database* en. Library Catalog: www.internal-displacement.org. https://www.internal-displacement.org/database.
2. Emanuel, K. Global Warming Effects on U.S. Hurricane Damage. en. *Weather, Climate, and Society* **3.** tex.ids= Emanuel2011a publisher: American Meteorological Society, 261–268. ISSN: 1948-8327. https://journals.ametsoc.org/wcas/article/3/4/261/799/Global-Warming-Effects-on-U-S-Hurricane-Damage (2011).
3. Eberenz, S., Lüthi, S. & Bresch, D. N. Regional tropical cyclone impact functions for globally consistent risk assessments. English. *Natural Hazards and Earth System Sciences* **21.** Publisher: Copernicus GmbH, 393–415. ISSN: 1561-8633. https://doi.org/10.5194/nhess-21-393-2021 (2021).
4. Knapp, K. R., Kruk, M. C., Levinson, D. H., Diamond, H. J. & Neumann, C. J. The International Best Track Archive for Climate Stewardship (IBTrACS): Unifying Tropical Cyclone Data. en. *Bulletin of the American Meteorological Society* **91.** Publisher: American Meteorological Society Section: Bulletin of the American Meteorological Society, 363–376. ISSN: 0003-0007, 1520-0477. https://journals.ametsoc.org/view/journals/bams/91/3/2009bams2755_1.xml (2010).
5. Chan, M. H. K., Wong, W. K. & Au-Yeung, K. C. Machine learning in calibrating tropical cyclone intensity forecast of ECMWF EPS. en. *Meteorological Applications* **28.** _eprint: https://onlinelibrary.wiley.com/doi/pdf/10.1002/met.2041, e2041. ISSN: 1469-8080. https://onlinelibrary.wiley.com/doi/abs/10.1002/met.2041 (2021).
6. Aijaz, S., Kepert, J. D., Ye, H., Huang, Z. & Hawksford, A. Bias Correction of Tropical Cyclone Parameters in the ECMWF Ensemble Prediction System in Australia. EN. *Monthly Weather Review* **147.** Publisher: American Meteorological Society Section: Monthly Weather Review, 4261–4285. ISSN: 1520-0493, 0027-0644. https://journals.ametsoc.org/view/journals/mwre/147/11/mwr-d-18-0377.1.xml (2019).
7. Owens, R. G. & Hewson, T. D. *ECMWF Forecast User Guide* en. Tech. rep. (ECMWF, 2018). https://www.ecmwf.int/en/elibrary/81307-ecmwf-forecast-user-guide.
